# Constitutive Photomorphogenic 1 Enhances ER Stress Tolerance in Arabidopsis

**DOI:** 10.3390/ijms221910772

**Published:** 2021-10-05

**Authors:** Chang Ho Kang, Eun Seon Lee, Ganesh M. Nawkar, Joung Hun Park, Seong Dong Wi, Su Bin Bae, Ho Byoung Chae, Seol Ki Paeng, Jong Chan Hong, Sang Yeol Lee

**Affiliations:** Division of Applied Life Sciences (BK21+) and Plant Molecular Biology and Biotechnology Research Center, Gyeongsang National University, Jinju 52828, Korea; jacobgnu69@gnu.ac.kr (C.H.K.); dmstjsl88@hanmail.net (E.S.L.); ganeshtnau@gmail.com (G.M.N.); jazzc@nate.com (J.H.P.); wsd3377@gmail.com (S.D.W.); dnflwlq7760@naver.com (S.B.B.); truedaisy@hanmail.net (H.B.C.); skpaeng@gmail.com (S.K.P.)

**Keywords:** endoplasmic reticulum (ER) stress, light signaling, unfolded protein response (UPR)

## Abstract

Interaction between light signaling and stress response has been recently reported in plants. Here, we investigated the role of CONSTITUTIVE PHOTOMORPHOGENIC 1 (COP1), a key regulator of light signaling, in endoplasmic reticulum (ER) stress response in Arabidopsis. The *cop1-4* mutant Arabidopsis plants were highly sensitive to ER stress induced by treatment with tunicarmycin (Tm). Interestingly, the abundance of nuclear-localized COP1 increased under ER stress conditions. Complementation of *cop1-4* mutant plants with the wild-type or variant types of *COP1* revealed that the nuclear localization and dimerization of COP1 are essential for its function in plant ER stress response. Moreover, the protein amount of ELONGATED HYPOCOTYL 5 (HY5), which inhibits bZIP28 to activate the unfolded protein response (UPR), decreased under ER stress conditions in a COP1-dependent manner. Accordingly, the binding of bZIP28 to the *BIP3* promoter was reduced in *cop1-4* plants and increased in *hy5* plants compared with the wild type. Furthermore, introduction of the *hy5* mutant locus into the *cop1-4* mutant background rescued its ER stress-sensitive phenotype. Altogether, our results suggest that COP1, a negative regulator of light signaling, positively controls ER stress response by partially degrading HY5 in the nucleus.

## 1. Introduction

Light signaling plays various roles throughout the life cycle of a plant by regulating diverse physiological processes such as seed germination, seedling photomorphogenesis, shade avoidance, phototropism, gravitropism, chloroplast movement, photoperiod responses, circadian rhythms, and flower induction [1]. Plants are equipped with at least four distinct families of photoreceptors: phytochromes (PHYs), which recognize red and blue light [1]; cryptochromes (CRYs) and phototropins (PHOTs), which perceive blue light [2]; and UV RESISTANCE LOCUS8 (UVR8), which recognizes ultraviolet (UV) light [3]. Among these photoreceptors, PHYA, PHYB, CRY1, and CRY2 play a major role in regulating photomorphogenesis in response to specific wavelengths of light. Photoreceptors activated by the perception of light signals inhibit CONSTITUTIVE PHOTOMORPHOGENIC 1 (COP1), which facilitates the accumulation of downstream positive regulators to regulate photomorphogenesis [4]. It has been suggested that the transcriptional responses to far-red, red, and blue light can be observed within 1 h of treatment, and these transcriptional responses correlate with the accumulation of ELONGATED HYPOCOTYL 5 (HY5), which is a basic leucine zipper (bZIP) transcription factor (TF) [5]. On the other hand, in the absence of light, COP1 mediates the ubiquitination and degradation of HY5 [6,7]. Interestingly, photoreceptors are also the targets of COP1 [8], while PHYs mediate the nuclear exclusion of COP1, thus repressing its activity [9]. Therefore, in the light signaling pathway, COP1 acts as a central negative regulator and as an E3 ligase, and its nuclear exclusion is a rate-limiting step.

Increasing evidence suggests that abiotic stresses such as high salt, heat, and oxidative stress can easily disturb the proper folding of proteins in the endoplasmic reticulum (ER), triggering ER stress [10,11,12]. In *Arabidopsis thaliana*, approximately one third of proteins are translocated to or secreted out of the cell membrane after biosynthesis. These proteins undergo folding and assembly processes in the ER to become functional before being translocated to suitable locations in the cell [10,11]. Under ER stress conditions, a type of response system, called the unfolded protein response (UPR), is activated to stop the stress and to re-establish ER homeostasis [13]. In other words, UPR is a type of response system that is activated by ER stress and enhances the expression of molecular chaperones, including the luminal binding protein (BiP), calreticulins (CRTs), and calnexin (CNX), and folding enzymes such as protein disulfide isomerase (PDI), in order to regain ER homeostasis [10,11]. In plants, proteolytic processing of the bZIP28 protein plays a critical role in the operation of UPR during ER stress. Under normal (stress-free) conditions, the inactivated form of bZIP28 is associated with the ER luminal membrane [14]. However, under ER stress, the terminal part of bZIP28 is cleaved by an unidentified proteinase and transferred to the Golgi apparatus, where the terminal region is cleaved once again by site-2 protease (S2P) [15]. Subsequently, the activated form of bZIP28 is transferred to the nucleus, where it induces the transcription of UPR genes [16].

If the level of misfolded proteins exceeds the ER quality control (ERQC) capacity, the ER-associated protein degradation (ERAD) machinery is activated to break down and remove the unfolded proteins [17]. Under prolonged ER stress, apparatuses involved in autophagy and programmed cell death (PCD) are activated to remove damaged cells [18,19].

Previous studies have shown that light intensity affects the expression of UPR marker genes, whereas the positive transcriptional regulator of light signaling, HY5, suppresses UPR; however, the molecular mechanism underlying these processes remains unclear [20]. In this study, we focus on roles of COP1, a central negative regulator of light signaling, during ER stress response [12]. Our results suggest that COP1 improves UPR, owing to its enrichment in the nucleus under ER stress conditions, followed by partial degradation of HY5.

## 2. Results

### 2.1. COP1 Mediated ER Stress Tolerance in Arabidopsis

**Figure 1:**
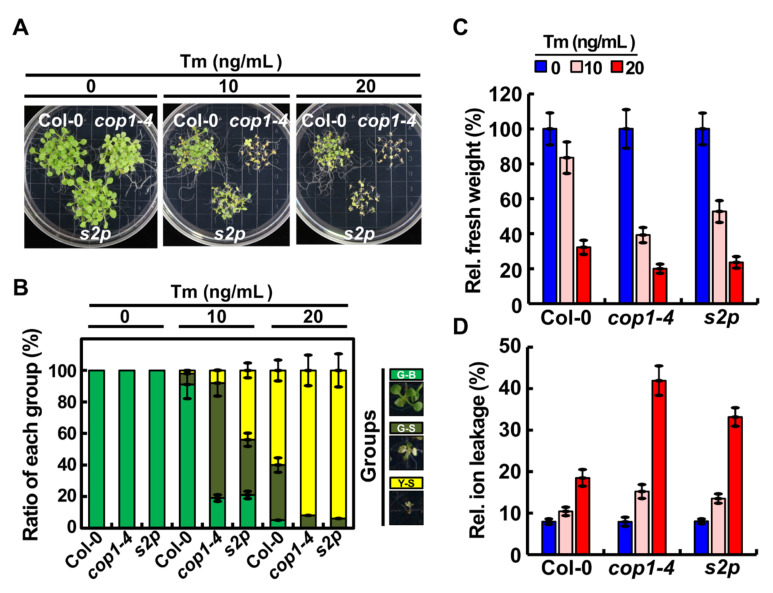
The *cop1-4* mutant was sensitive to ER stress. (**A**) Phenotypes of 2-week-old WT (Col-0), *cop1-4*, and *s2p* seedlings grown on MS medium containing 0, 10, or 20 ng/mL tunicamycin (Tm). (**B**) Percentage of Tm-treated plants of each genotype in each of the three classes, green-big (G-B), green-small (G-S), and yellow-small (Y-S) plants, depending on their sensitivity to Tm. Data represent mean ± standard deviation (SD; *n* = 3). (**C**,**D**) Relative fresh weight (**C**) and electrolyte leakage (**D**) of plants treated with Tm, as indicated in (**A**). Data represent mean ± SD (*n* = 3).

To explore the crosstalk between light signaling and ER stress response in plants, we focused on COP1, which is known as a key regulator of light signaling in plants [4], and investigated its role in ER stress response. Since the null mutation of *COP1* is lethal to plants [21,22], we used the *cop1-4* mutant, which carries a point mutation in the *COP1* coding sequence; this point mutation changes the CAA codon (which corresponds to the Gln-283 residue) to the UAA stop codon, resulting in truncated COP1 protein, which contains only 282 N-terminal amino acid residues [23]. We first verified the transcript level of *COP1* in *cop1-4* mutant and wild-type (WT; Col-0) plants by quantitative real-time polymerase chain reaction (qRT-PCR). Interestingly, the transcript level of *COP1* was markedly lower in the *cop1-4* mutant than in the WT (Figure S1), which is consistent with the previous finding that point mutation of *COP1* in the *cop1-4* mutant decreases the level of COP1 protein [23]. Next, to investigate the role of COP1 in ER stress response, seeds of *cop1-4*, *s2p* T-DNA mutant (control) [24], and WT genotype were germinated on Murashige and Skoog (MS) media containing 0, 10, or 20 ng/mL tunicamycin (Tm), and seedlings were grown under a long-day (LD) photoperiod (16 h light/8 h dark) for 2 weeks. The results showed that *cop1-4* seedlings, such as *s2p* seedlings, were highly sensitive to the Tm-induced ER stress (Figure 1A). On the basis of sensitivity, the seedlings were grouped into three classes: green-big (G-B), green-small (G-S), and yellow-small (Y-S). Similar to *s2p,* a greater number of *cop1-4* seedlings grouped in the Y-S and G-S classes under ER stress compared with the WT (Figure 1B). ER stress responses of WT, *s2p*, and *cop1-4* seedlings were compared by rating their fresh weight and electrolyte leakage when grown on media containing Tm (Figure 1C,D). The results showed that the fresh weight of *cop1-4* seedlings was almost half that of WT seedlings in the presence of 10 ng/mL Tm (Figure 1C). Additionally, the electrolyte leakage of *cop1-4* seedlings was much higher than that of WT seedlings under ER stress conditions (Figure 1D). Moreover, like *cop1-4* seedlings, *cop1-6* mutant seedlings [23] were also highly sensitive to Tm (Figure S2). Moreover, when treated with 5 μg/mL Tm for 6 hours, growth inhibition was severer in *cop1-4* and *cop1-6* mutants compared to that in WT seedlings (Figure S3). Overall, these results indicate that COP1 promotes ER stress tolerance in Arabidopsis.

### 2.2. COP1 Was Enriched in the Nucleus under ER Stress Conditions

**Figure 2:**
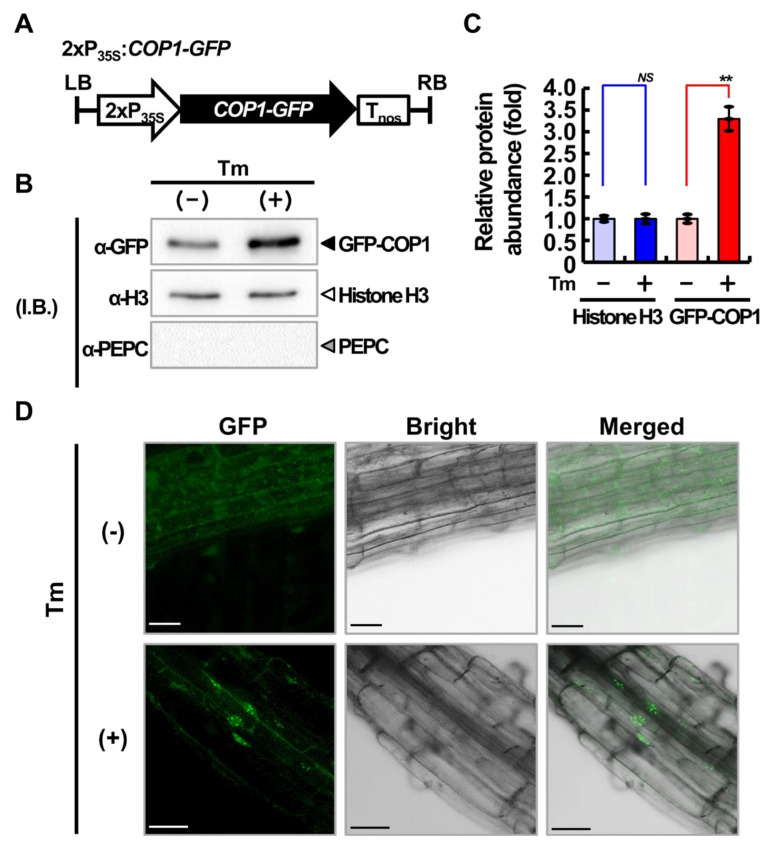
Nuclear enrichment of COP1 under ER stress. (**A**) Schematic representation of the construct used in the experiments. P_35S_ and T_nos_ represent the cauliflower mosaic virus (CaMV) *35S* promoter and *nopaline synthase* (*NOS*) terminator. (**B**) Comparison of the COP1-GFP fusion protein abundancy in the nucleus of Tm-treated (+) and untreated (−) transgenic plants harboring the construct depicted in (**A**). Total protein was extracted from transgenic plants, and the nuclear extract was fractionated. The COP1-GFP protein was detected by immunoblotting with anti-GFP antibody, and the nuclear specificity was determined using antibodies against marker proteins (anti-Histone H3, nucleus; and anti-PEPC, cytosol). (**C**) Quantification of the relative abundance (fold enrichment) of the nuclear-localized COP1-GFP protein. The abundance of histone H3 was determined as a control. Data represent mean ± SD of three independent biological replicates. Asterisks indicate statistically significant differences (Student’s *t*-test; ** *p* < 0.01; *NS*, not significant). (**D**) Translocation of COP1 protein in response to the Tm treatment. The root epidermis of transgenic plants overexpressing *COP1-GFP* were subjected to confocal microscopy before (−Tm) or after (+Tm) treatment with 5 μg/mL Tm for 6 h. Scale bars = 20 μm.

Under visible light, a well-known inhibitory mechanism of COP1 involves its export from the nucleus by light-activated photoreceptors [25,26]. On the other hand, in the dark, COP1 acts as a repressor of photomorphogenesis by directly targeting photoreceptors and TFs that promote light signaling via the 26S proteasomal degradation pathway [7,21]. Moreover, COP1 is exported from the nucleus to the cytoplasm in response to cold stress, heat shock, and high salinity [27,28,29]. Therefore, we examined the subcellular localization of COP1 under ER stress conditions. Total proteins were extracted from Tm-treated or untreated 10-day-old transgenic plants overexpressing the *COP1-green fluorescent protein* (*GFP*) gene fusion [30], and their nuclear extracts were fractionated (Figure 2). The level of nuclear COP1-GFP protein was determined by immunoblotting with anti-GFP antibody (Figure 2B). The relative abundance of nuclear-localized COP1-GFP protein in Tm-treated plants was quantified and compared with that in untreated plants (Figure 2C). The degree of nuclear fraction enrichment was determined with a specific antibody for histone H3 (nuclear marker). The level of nuclear-localized COP1-GFP was higher in Tm-treated plants than that in untreated plants. These results were further confirmed by confocal microscopy. The root epidermis of transgenic plants overexpressing *COP1-GFP* were subjected to confocal microscopy before (−Tm) or after (+Tm) treatment with 5 μg/mL Tm for 6 h. The results showed that the green fluorescence signal of the COP1-GFP fusion protein was enriched in the nucleus of Tm-treated plants (Figure 2D). Altogether, our results suggest that COP1 is enriched in the nucleus under ER stress conditions.

### 2.3. COP1 Nuclear Localization and Dimerization Were Essential for Its Role in ER Stress Tolerance

COP1 protein has three conserved structural domains: RING finger domain, coiled-coil domain, and WD40 repeat domain [23,25,31,32,33]. The RING finger domain of COP1 is responsible for its ubiquitin E3 ligase activity [25], which is required for the degradation of target proteins [7]. The coiled-coil domain of COP1 is required for its homodimerization and consequently activation, while the WD40 repeat domain mediates its interaction with different protein substrates. Additionally, COP1 harbors a bipartite nuclear localization signal (NLS) and a cytoplasmic localization signal (CLS), which are involved in regulating the localization of COP1 in response to light [25]. To determine the function of COP1 in the nucleus or cytoplasm, the CLS or NLS was mutated, as described previously, to generate *COP1-GFP* mutant alleles (Figure S4A) [25,32]. Mutant COP1 protein harboring substitutions of four conserved leucine (Leu) residues to alanine (Ala) at amino acid positions 103, 104, 107, and 108 was designated as COP1^L105A^, whereas that harboring substitutions of another four conserved Leu residues to Ala at positions 167, 168, 171, and 174 was designated as COP1^L170A^ [25,32]. In addition, mutant COP1 protein carrying substitutions of basic amino acid residues arginine (Arg) and lysine (Lys) to serine (Ser; positions 294 and 314) and threonine (Thr; positions 296 and 312), respectively, was designated as COP1^MUT^ [25,32]. The *COP1-GFP*, *COP1^L105A^-GFP*, *COP1^L170A^-GFP*, and *COP1^MUT^-GFP* fusions were transiently expressed in tobacco (*Nicotiana benthamiana*) leaves, and the subcellular localization of the corresponding fusion proteins was examined by confocal microscopy (Figure S3b). A construct expressing the *red fluorescence protein* (*RFP*) gene fused to the NLS (*NLS-RFP*) was used as a nuclear marker (control). COP1^L105A^-GFP and COP1^L170A^-GFP localized mainly in the nucleus, whereas COP1^MUT^-GFP localized only to the inclusion bodies in the cytosol (Figure S4B) [25,32]. Next, we generated *cop1-4* complementation lines expressing the *COP1-GFP*, *COP1^L105A^-GFP*, *COP1^L170A^-GFP*, and *COP1^MUT^-GFP* fusions. At least two lines per construct were selected, and levels of recombinant proteins were determined in all selected lines (Figure S5A). It is well known that *cop1-4* is a weak mutant allele of *COP1*, showing constitutive photomorphogenic development even in the dark. The *cop1-4* mutant shows short hypocotyl and open cotyledons in the dark. To investigate the functionality of *COP1* mutant alleles, we grew all *cop1-4* complementation lines under continuous dark, and their developmental phenotypes were carefully observed. The results showed that the complementation lines expressing *COP1-GFP* and *COP1^L105A^-GFP* well rescued the growth defects of the *cop1-4* mutant in both light and dark conditions (Figure S5B, C) [25,32,34]. Interestingly, the *COP1^MUT^/cop1-4* lines showed long hypocotyl and open cotyledons/hook in the dark. This means that at least the hypocotyl elongation phenotype in the dark has been complemented in *COP1^MUT^/cop1-4* line.

**Figure 3:**
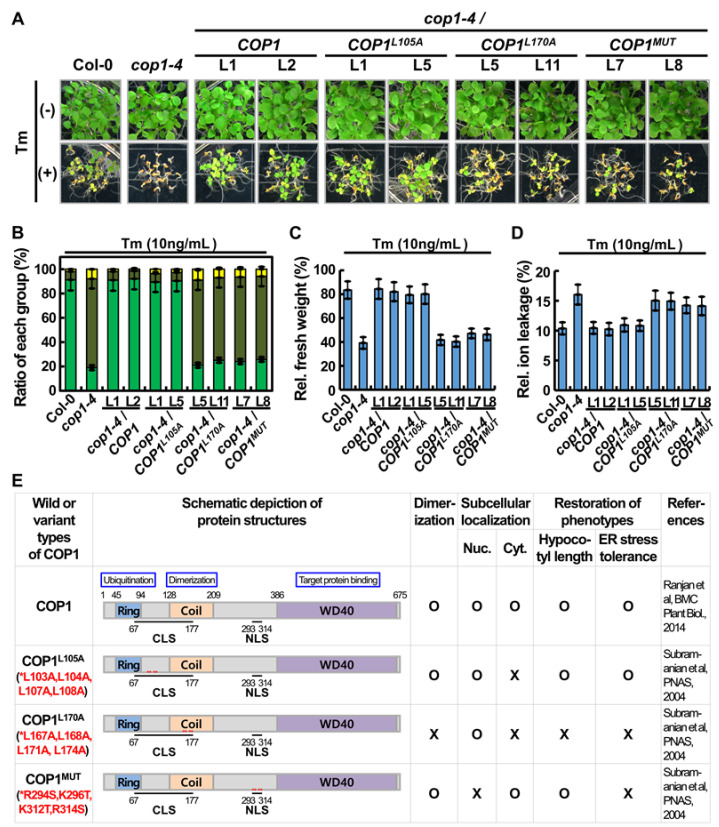
Phenotypic analyses of diverse *cop1-4* complementation lines under ER stress. (**A**) Representative photographs of seedlings of the WT (Col-0), *cop1-4* mutant, and selected *cop1-4* complementation lines expressing *COP1*, *COP1^L105A^*, *COP1^L170A^*, and *COP1^MUT^* grown on the MS media supplemented with 10 ng/mL Tm (+Tm) or no Tm (−Tm) under an LD photoperiod for 2 weeks. (**B**) Percentage of Tm-treated plants of each genotype in each of the three classes, G-B, G-S, and Y-S, depending on their sensitivity to Tm. Data represent mean ± SD (*n* = 3). (**C**,**D**) Relative fresh weight (**C**) and electrolyte leakage (**D**) of plants of each genotype treated with 10 ng/mL Tm. Data represent mean ± SD (*n* = 3). (**E**) Tabulation of the properties of COP1, COP1^L105A^, COP1^L170A^, and COP1^MUT^ proteins. The RING domain (Ring) is important for the ubiquitination of target proteins. The coiled-coil motif (Coil) is important for COP1 dimerization. The C-terminal WD40 domain (WD40) plays an important role in the binding of COP1 to its target proteins. Red asterisks indicate the locations of amino acid substitutions in the three COP1 variants. The ability of each protein to localize to the nucleus (Nuc.) or cytosol (Cyt.), to dimerize, to restore the hypocotyl elongation phenotype under dark condition, and to rescue the ER stress-sensitive phenotype of the *cop1-4* mutant are indicated by O (Yes) or X (No).

Because *cop1-4* mutant plants were sensitive to ER stress (Figure 1), seeds of all *cop1-4* complementation lines, the WT, and *cop1-4* mutant were germinated in the presence or absence of Tm, and the sensitivity of seedlings to ER stress was analyzed (Figure 3A). The *cop1-4* mutant seedlings displayed sensitive phenotypes in the Tm-induced ER stress condition compared with WT seedlings, as expected. However, complementation lines expressing *COP1-GFP* and *COP1^L105A^-GFP* successfully rescued the ER stress-sensitive phenotypes of the *cop1-4* mutant. By contrast, complementation lines expressing *COP1^L170A^-GFP* and *COP1^MUT^-GFP* failed to rescue the ER stress-sensitive phenotypes of the *cop1-4* mutant. On the basis of sensitivity to the Tm-induced ER stress, the seedlings were grouped into the G-B, G-S, and Y-S classes (Figure 3B). More than 90% of the complementation lines expressing *COP1-GFP* and *COP1^L105A^-GFP* were grouped in the G-B class, whereas <40% of the complementation lines expressing *COP1^L170A^-GFP* and *COP1^MUT^-GFP* were grouped in the G-B class, implying that COP1^L170A^ and COP1^MUT^ could not rescue the ER stress sensitive phenotype of the *cop1-4* mutant. ER stress responses of *cop1-4* complementation lines were confirmed by comparing their fresh weight and electrolyte leakage when grown in the presence or absence of Tm (Figure 3C,D). The results showed that fresh weights of complementation lines expressing *COP1-GFP* and *COP1^L105A^-GFP* recovered close to that of WT seedlings grown in the presence of Tm (Figure 3C). Additionally, electrolyte leakage of *cop1-4* mutant seedlings and complementation lines expressing *COP1^L170A^-GFP* and *COP1^MUT^-GFP* was much higher than that of WT plants and complementation lines expressing *COP1-GFP* and *COP1^L105A^-GFP* (Figure 3D). These data are summarized in Figure 3E.

### 2.4. COP1 Mediated Partial Degradation of HY5 under ER Stress Conditions

**Figure 4:**
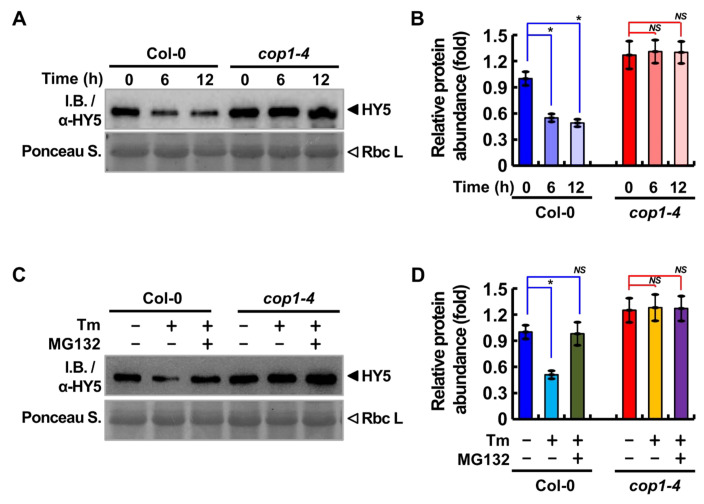
COP1-mediated partial degradation of HY5 under ER stress. (**A**) Time-dependent degradation of HY5 in WT (Col-0) and *cop1-4* plants after Tm treatment. Total proteins were extracted from 10-day-old seedlings treated with 5 μg/mL Tm for 0, 6, and 12 h through vacuum infiltration, and HY5 was detected by immunoblotting with anti-HY5 antibody. (**B**) Quantification of the relative abundance of HY5 protein in the blots shown in (**A**). The amount of HY5 detected in WT and *cop1-4* seedlings at the 6 and 12 h time points was normalized relative to that detected in WT seedlings at the 0 h time point (control condition). (**C**) Confirmation of proteasome-mediated HY5 degradation. Total proteins were extracted from 10-day-old WT (Col-0) and *cop1-4* seedlings subjected to treatment with (+) or without (−) 5 μg/mL Tm for 6 h, followed by treatment with (+) or without (−) 50 μM MG132. The HY5 protein was detected by immunoblotting analysis with anti-HY5 antibody. (**D**) Quantification of the relative abundance of HY5 protein samples shown in (**C**). Data at each time point were normalized relative to the control condition (Col-0, −Tm, −MG132). In (**B**,**D**), data represent ± standard error of mean (SEM) of three independent biological replicates. Asterisks indicate statistically significant differences (Student’s *t*-test; * *p* < 0.05; *NS*, not significant).

HY5 is known to regulate the light-responsive transcriptional cascade [35,36]. In the dark, COP1 translocates from the cytosol to the nucleus and mediates the degradation of HY5 by the 26S proteasome [7]. Recently, HY5 was shown to function as a negative regulator of ER stress response [20]. In this study, COP1 was enriched in the nucleus under ER stress conditions (Figure 2). To confirm whether COP1 regulates the stability of HY5 under ER stress conditions, we germinated WT and *cop1-4* seeds on MS medium and treated them with 5 μg/mL Tm for 0, 6, and 12 h through vacuum infiltration (Figure 4A,B). Then, the amount of HY5 protein was assessed by immunoblotting with anti-HY5 antibody. The results showed that the HY5 protein level in Tm-treated WT seedlings decreased gradually in a time-dependent manner. Contrastingly, the level of HY5 was maintained in *cop1-4* seedlings, regardless of the Tm treatment, suggesting that COP1 mediates HY5 degradation under ER stress conditions. To confirm this result, we simultaneously treated Tm-treated seedlings with MG132, a chemical inhibitor of the 26S proteasome, and the level of HY5 was analyzed (Figure 4C,D). The results showed that HY5 degradation in Tm-treated WT seedlings was inhibited by MG132. Furthermore, when HY5 degradation under ER-stress condition was assessed in *cop1-4* complementation lines, HY5 protein level in the Tm-treated seedlings was lower in *COP1*/*cop1-4* and *COP1^L105A^*/*cop1-4* compared to that in *COP1^L170A^*/*cop1-4* and *COP1^mut^*/*cop1-4* (Figure S6).

### 2.5. COP1 Facilitated the Binding of bZIP28 to ER Stress Response Element (ERSE) under ER Stress

**Figure 5:**
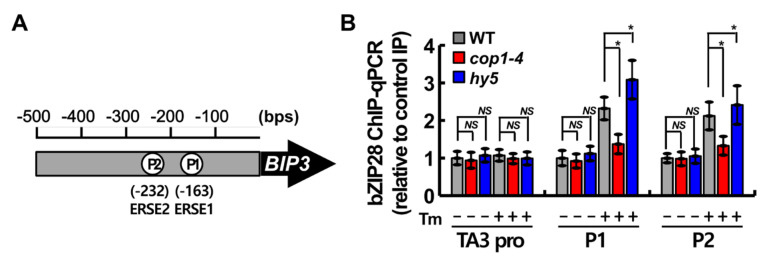
Analysis of the binding of bZIP28 to the *BIP3* promoter in WT, *cop1-4*, and *hy5* plants by ChIP assay. (**A**) Schematic representation of the *BIP3* promoter showing the location of the two ERSE motifs. P1 and P2 represent the respective primer positions used for ChIP-quantitative PCR (qPCR). (**B**) ChIP-qPCR analysis of the binding of bZIP28 to the *BIP3* promoter. ChIP assays were performed using 10-day-old WT (Col-0) and *cop1-4* seedlings treated with Tm (+) or dimethylsulfoxide (DMSO; control) (−) for 6 h. DNA–protein complexes were immunoprecipitated using antibodies against bZIP28 and rabbit IgG (negative control). ChIP DNA was quantified by qRT-PCR, with primers specific to the ERSE motifs (P1 and P2) and *TA3* promoter (control). Data represent mean ± SEM (*n* = 3 technical replicates). Asterisks indicate statistically significant differences (Student’s *t*-test; * *p* < 0.05; *NS*, not significant).

According to our previous study [20], HY5 negatively regulates the UPR response by competing with bZIP28 to bind to the ERSE motifs in target gene promoters, and the binding of HY5 to the ERSE motifs is significantly reduced under ER stress. Therefore, to investigate how COP1 regulates the UPR response, we compared the occupancy of the ERSE sequences in the *BIP3* promoter by bZIP28 in WT, *cop1-4*, and *hy5* plants by chromatin immunoprecipitation (ChIP) experiments (Figure 5A). After the immunoprecipitation of protein–DNA complexes using anti-bZIP28 antibody, the DNA fragments were quantified by qRT-PCR (Figure 5B). The *TA3* promoter was used as a negative control [37]. In Tm-treated WT plants, bZIP28 showed remarkably greater occupancy at P1 and P2 regions in the *BIP3* promoter, which contains ERSE1 and ERSE2 motifs, respectively, than at the negative control site. Interestingly, in the Tm treatment, bZIP28 showed significantly lower occupancy at the P1 and P2 regions in the *cop1-4* mutant and higher occupancy in the *hy5* mutant compared with the WT (Figure 5B). These results indicate that COP1 improves, but HY5 inhibits, the association of bZIP28 with the ERSE motifs in the *BIP3* promoter in vivo.

### 2.6. hy5 Rescued the ER Stress-Sensitive Phenotype of cop1-4

**Figure 6:**
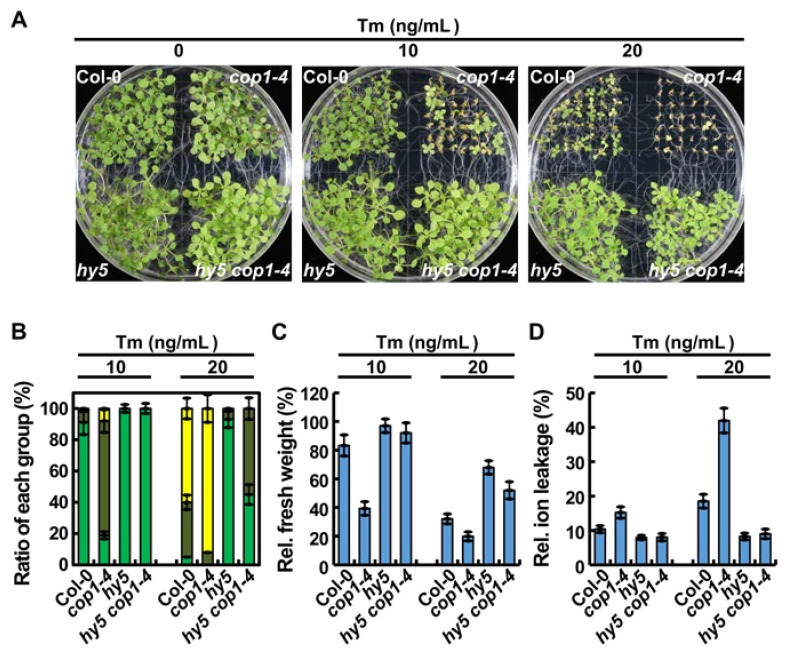
The *hy5* mutant locus confers ER stress resistance in *cop1-4* plants. (**A**) Phenotypes of 2-week-old WT (Col-0), *cop1-4*, *hy5*, and *hy5 cop1-4* seedlings grown on MS medium containing 0, 10, or 20 ng/mL Tm. (**B**) Percentage of plants of each genotype in the three classes, G-B, G-S, and Y-S, depending on their sensitivity to Tm. Data represent mean ± SD (*n* = 3). (**C**,**D**) Relative fresh weight (**C**) and electrolyte leakage (**D**) of plants treated with Tm, as indicated in (**A**). Data represent mean ± SD (*n* = 3).

Our results showed that COP1 enhances ER stress tolerance by partially degrading HY5, which is a negative regulator of the UPR [20]. This prompted us to investigate the genetic link between *HY5* and *COP1* in ER stress response. To conduct this experiment, we crossed *hy5* and *cop1-4* plants to generate the *hy5 cop1-4* double mutant (Figure S1), and a stable homozygous double mutant line was identified. Then, WT, *cop1-4*, *hy5*, and *hy5 cop1-4* plants were grown on MS media supplemented with 0, 10, and 20 ng/mL Tm, and their growth phenotypes were compared (Figure 6A). The *hy5* mutant showed a strong resistant phenotype, as expected, as evident from the greater number of *hy5* plants than WT plants in the G-B class under ER stress conditions (Figure 6B). Interestingly, compared with *cop1-4* plants, the number of *hy5 cop1-4* double mutant plants was greater in the G-B class and lower in the Y-S class under ER stress conditions (Figure 6B). Additionally, to confirm the differences between ER stress responses of the four genotypes, we compared the fresh weight and electrolyte leakage of the seedlings grown on media supplemented with 0, 10, and 20 ng/mL Tm (Figure 6C,D). The results showed that the fresh weight of *hy5 cop1-4* seedlings was 2.3- and 3.5-fold higher than that of *cop1-4* seedlings grown in the presence of 10 and 20 ng/mL Tm, respectively (Figure 6C). Additionally, in the presence of Tm, the electrolyte leakage of *hy5 cop1-4* seedlings was much less than that of WT and *cop1-4* seedlings (Figure 6D). Altogether, our data imply that introduction of the *hy5* locus in the *cop1-4* mutant background restores ER stress resistance. Additionally, these results confirmed that COP1 targets HY5 under ER stress conditions [5]. The opposite phenotype of *hy5* and *cop1-4* mutant plants suggests that HY5 and COP1 work antagonistically in ER stress response.

## 3. Discussion

**Figure 7:**
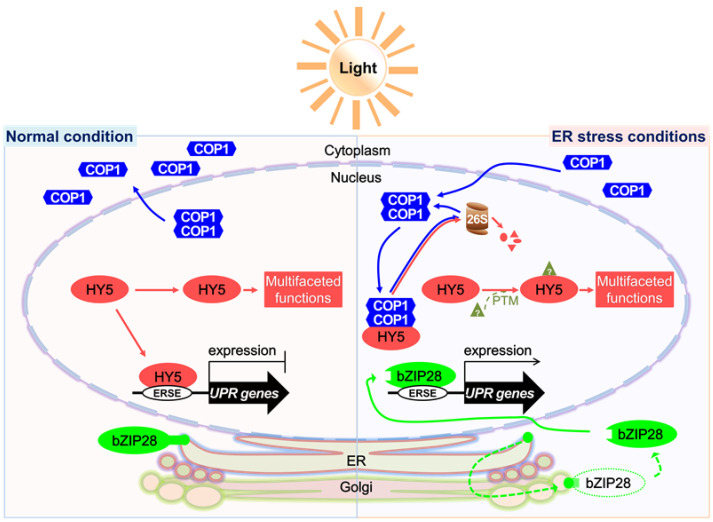
Model depicting the role of COP1 in ER stress. Under normal light conditions, activated photoreceptors perceive the light signals and mediate the translocation of COP1 from the nucleus to the cytoplasm, while simultaneously promoting downstream light signaling components such as HY5, which displays multifaceted roles. HY5 also occupies the promoters of UPR genes and suppresses their expression. Under ER stress conditions, COP1 is enriched in the nucleus, where it mediates the partial degradation of HY5 via the 26S proteasome. This in turn allows the truncated bZIP28 protein to bind to the promoters of UPR genes and to activate their expression, thus enhancing the ER stress resistance of plants. Blue, red and green lines express movements of COP1, HY5 and bZIP28, respectively.

Considering our results, we propose a hypothetical model that explains the role of COP1 in the UPR as a key component of the light signaling pathway (Figure 7). Under unstressed conditions, light is perceived by photoreceptors, which promote the export of COP1 from the nucleus to the cytoplasm [1,9]. Activated photoreceptors transduce signals to the downstream target protein HY5 to mediate the photomorphogenesis of plants [35,36]. HY5 binds to the promoters of UPR marker genes to repress their expression to the basal level [20]. The UPR regulated by bZIP28 is inactive in this state [15,16]. However, exposure to environmental stress promotes the accumulation of unfolded proteins in plant cells, which induces ER stress [11,38]. This activates the UPR to improve the protein folding capacity of the ER and to alleviate ER stress [10,14]. The UPR regulated by bZIP28 is activated by the truncation of bZIP28 and by the subsequent translocation of the activated protein to the nucleus [15,16]. Additionally, COP1, which was located in the cytoplasm under normal light conditions, is translocated to the nucleus under ER stress conditions, where it degrades approximately 50% of the HY5 protein through the 26S proteasome machinery (Figure 2 and Figure 4), thus facilitating the binding of the activated bZIP28 protein to the promoters of UPR genes to activate their expression (Figure 5) [20].

In the current study, we used two *cop1* mutant alleles to investigate the role of COP1 in ER stress response, *cop1-4*, which encodes a truncated protein of 282 amino acids, and *cop1-6*, which encodes COP1 protein harboring the five-amino acid insertion; these mutants were used since the complete loss of COP1 is lethal [22,23,39]. Interestingly, both *cop1-4* and *cop1-6* mutants were remarkably sensitive to the Tm-induced ER stress (Figure 1 and Figure S2). The ER stress-sensitive phenotype of *cop1-4* was fully rescued by complementation with constructs expressing *COP1* and *COP1^L105A^* but not by those expressing *COP1^L170A^* and *COP1^MUT^* (Figure 3, Figures S3 and S4). These results are consistent with those of previous studies, which showed that the nuclear localization of COP1 is important for regulation of its target proteins, which is needed to suppress photomorphogenesis [25,32,40,41]. Despite the nuclear localization of the COP1^L170A^, complementation of the *cop1-4* mutant with *COP1^L170A^* failed to rescue its ER stress-sensitive phenotype and to normalize its photomorphogenic response (Figure 3, Figures S3 and S4). Previously, it was suggested that the L170A substitution disrupts the coiled-coil domain of COP1, which inhibits its homodimerization and reduces its functionality [25,32]. This implies that the nuclear localization and intermolecular dimerization are important properties of COP1 required not only for developmental regulation but also for ER stress tolerance. Accumulating evidence suggests that not only light but also other factors such as hormones or temperature cues can regulate the subcellular localization and functionality of COP1 [25,27,28,29]. For example, COP1 activity is negatively regulated by cytokinin and positively regulated by gibberellic acid (GA) [42,43]. Similarly, cold and heat stresses can trigger the export of COP1 from the nucleus into the cytosol, which in turn elevates the level of HY5 in the nucleus [28,29]. In the current study, we showed that COP1 is enriched in the nucleus under ER stress, where it decreases the level of its downstream target protein, HY5 (Figure 2 and Figure 4). The detailed mechanism of how COP1 is enriched in the nucleus under ER stress conditions will be revealed in future studies.

In plants treated with Tm, approximately 50% of the HY5 protein was degraded by COP1 in the nucleus. This implies that COP1 controls the UPR positively by destabilizing HY5, a well-known negative regulator of the UPR [20]. Moreover, we showed that the occupation of the *BIP3* promoter by bZIP28 was significantly less in *cop1-4* plants than in WT plants in the Tm treatment. Interestingly, even in the Tm treatment, approximately 50% of HY5 remained intact (Figure 4), which suggests that much of HY5 is stabilized by an unknown mechanism functional under ER stress. A recent study reported that SUPPRESSOR OF PHYA-105 1 (SPA1) fine-tunes the stability and activity of HY5 via its phosphorylation to ensure proper photomorphogenesis [44]. It would be interesting to examine if SPA kinases are involved in ER stress response through the phosphorylation of HY5. The proper integration of light signaling and ER stress response can enhance plant survival under fluctuating environmental conditions.

In the dark condition, COP1 suppresses photomorphogenesis by translocating from the cytosol to the nucleus, which in turn results in the degradation of HY5 [5,8]. In this study, HY5 was partially degraded by COP1 under the ER stress condition (Figure 3), suggesting that COP1 mediates ER stress response through HY5 [38]. To examine the genetic interaction between *COP1* and *HY5*, we experimentally assessed ER stress phenotypes of the *hy5 cop1-4* double mutant while considering the contrasting ER stress sensitivities of *cop1-4* and *hy5* single mutants. The Tm-sensitivity of the *cop1-4* mutant was successfully recovered by the introduction of the *hy5* mutant allele; however, the *hy5 cop1-4* double mutant was not as Tm-tolerant as the *hy5* single mutant (Figure 5). COP1 acts as a negative regulator of light signaling through its E3 ligase activity, which promotes the ubiquitination and degradation of positive regulators of light signaling including HY5 and members of the B-box (BBX) family proteins [45,46,47]. Moreover, it is well known that COP1 regulates stress responses through its E3 ligase activity [33]. The resistance protein HRT, which induces hypersensitive response (HR) upon Turnip crinkle virus (TCV) infection, is degraded by COP1 [48]. The protein level and activity of AtSIZ1, an E3 SUMO ligase, are also directly regulated by COP1, and AtSIZ1-mediated plant abiotic stress responses are controlled by COP1 [49,50]. These findings suggest that COP1 is involved in the regulation of other target proteins as well as HY5 under ER stress conditions, which may explain why the *hy5 cop1-4* double mutant is not as tolerant to ER stress as the *hy5* single mutant.

## 4. Materials and Methods

### 4.1. Plant Materials and Growth Conditions

*Arabidopsis thaliana* ecotype Columbia (Col-0) was used as the WT in this study. All T-DNA insertion mutants and transgenic lines used in this study were also in Col-0 background. Homozygous T-DNA mutant *hy5* (SALK_096651C) was obtained from the Arabidopsis Biological Resource Center (ABRC; Ohio State University, Columbus, OH, USA). The *hy5 cop1-4* double mutant plants were generated by crossing *hy5* (female parent) with *cop1-4* (male parent), and homozygous lines were confirmed by PCR. Seeds were sown on full-strength MS medium (Duchefa Biochemie B.V., Haarlem, the Netherlands) containing 2% (*w*/*v*) sucrose and 0.25% (*w*/*v*) phytagel (Sigma-Aldrich, St. Louis, MO, USA) (pH 5.7). The plates were incubated in the dark at 4 °C for 3 days for the stratification of seeds and then transferred to an environmentally controlled growth chamber maintained at 22 °C, LD photoperiod, and 100–120 μmol m^−2^ s^−1^ photosynthetic flux with white light.

### 4.2. ER Stress Treatment and Phenotypic Analysis

To conduct ER stress treatment, we sowed seeds in MS medium containing 0–20 ng/mL Tm (Sigma-Aldrich). The plates were incubated in the dark at 4 °C for 3 days for the stratification of seeds, and then transferred to a growth chamber. To conduct phenotypic analysis, we grew plants in the presence or absence of Tm under an LD photoperiod for 2 weeks. The survival rate of plants was assessed in at least three replicates using 40–50 seeds per replicate. Seedlings were grouped into G-B, G-S, and Y-S classes, according to the leaf color and plant size [17,38]. The number of plants in each class was counted, and the percentage was calculated. Plants subjected to phenotypic analysis were also used for fresh weight analysis. Electrolyte leakage was assessed using 2-week-old seedlings treated with Tm, as described previously [51]. Briefly, five seedlings treated or untreated with Tm were immersed in de-ionized water for 3 h, and electrical conductivity of the solution was determined using the Orion 3 star conductometer (Thermo Electron Cooperation, Beverly, MA, USA). After autoclaving samples at 121 °C for 15 min, we re-measured electrical conductivity to obtain the total amount of ions in the cell. Electrolyte ion leakage was expressed as a percentage of the ratio of electrical conductivity before autoclaving to that after autoclaving. This experiment was repeated three times, with similar results.

### 4.3. Plasmid Construction and Plant Transformation

*COP1* coding sequence minus the stop codon was PCR-amplified from Col-0 cDNA using COP1_attB1_F and COP1_attB2_R primers. The gel-eluted PCR product was used for a second round of PCR amplification with the attB1 and attB2 primers. The PCR product was cloned into pDONR221 using the BP reaction kit (Invitrogen, Carlsbad, CA, USA), according to the manufacturer’s instructions, to generate the pDONR-COP1 construct. *COP1^L105A^*, *COP1^L170A^*, and *COP1^MUT^* were generated by amplifying the pDONR-COP1 DNA (as a template) with specific primer sets (Table S1) using the QuickChange™ Site-Directed Mutagenesis Kit (Stratagene, La Jolla, CA, USA), as described previously [52]. Then, *COP1* (WT), *COP1^L105A^*, *COP1^L170A^*, and *COP1^MUT^* were cloned into the pMDC85 vector [53], obtained from ABRC (CD3-744), using an LR reaction kit (Invitrogen), according to the manufacturer’s instructions, to generate pMDC85-COP1, pMDC85-COP1^L105A^, pMDC85-COP1^L170A^, and pMDC85-COP1^MUT^ constructs, respectively. All of the above constructs were transformed into *Agrobacterium tumefaciens* strain GV3101, which was then used to transform Arabidopsis Col-0 plants using standard protocols [54]. Transgenic lines were selected on plates containing 30 μg/mL hygromycin (Duchefa) and 250 μg/mL cefotaxime (Duchefa), and were confirmed by immunoblot analysis.

### 4.4. Transient Tobacco Expression Assay

*A. tumefaciens* strains GV3101 transformed with the test constructs were grown in LB medium supplemented with 10 mM MES, 20 μM acetosyringone, and appropriate antibiotics. Cells were collected by centrifugation, washed twice with infiltration solution (10 mM MgCl_2_, 10 mM MES, and 100 μM acetosyringone), and then mixed with cells harboring the P19 silencing suppressor plasmid, which were prepared separately. The cell suspensions were adjusted to an optical density (OD_600_) of 0.5 in infiltration solution. Leaves of 4-week-old tobacco (*Nicotiana benthamiana*) plants were co-infiltrated with the desired combination of cultures and *NLS-RFP* (nuclear marker control), and plants were incubated for 3 days. The expression of recombinant proteins was monitored at various time points after transformation using a confocal laser scanning microscope (FV1000 configuration with IX81 microscope; Olympus, Tokyo, Japan). GFP and RFP signals were detected using the U-MWB2 (exciter, BP 460-490; dichroic, DM505; emitter, LP 520) and U-MWU2 (exciter, HQ480/40; dichroic, Q505lp; emitter, HQ535) mirror units, respectively.

### 4.5. Subcellular Localization

Five-day-old transgenic plants overexpressing *COP1-GFP* were treated with or without 5 μg/mL Tm for 6 h. The subcellular localization of COP1-GFP was examined in transgenic roots using a confocal laser scanning microscope (FV1000 configuration with IX81 microscope; Olympus).

### 4.6. Nuclear Fractionation Experiment

To confirm the nuclear enrichment of COP1 under ER stress, we treated 10-day-old transgenic Arabidopsis plants overexpressing *COP1-GFP* with 0 or 5 μg/mL Tm for 6 h. Nuclear protein extraction was performed using the CelLytic PN extraction kit (Sigma-Aldrich). To isolate nuclei, we ground 300 mg of seedlings into a fine powder in liquid nitrogen using a prechilled mortar and pestle. The ground samples were mixed with 0.5 mL of 1× nuclear isolation buffer (NIB; from Sigma-Aldrich). The suspension was filtered through a miracloth (pore size: 22–25 μm) into a 1.5 mL Eppendorf tube. Organelles including nuclei were pelleted after centrifugation at 1260× *g* for 10 min. The pellet was completely resuspended in 0.5 mL of 1× NIBA (NIB buffer containing protease inhibitor cocktail), and organelle membranes were lysed by adding 10% Triton X-100 to a final concentration of 0.3%. To obtain a semi-pure preparation of nuclei, we applied the lysates to the top of a 0.8 mL cushion of 1.5 M sucrose with 1× NIBA in 1.5 mL Eppendorf tubes. The sample was centrifuged at 12,000× *g* for 10 min, and the upper phase and sucrose cushion were removed. The pellet was washed twice with 1× NIBA. The nuclear pellet was resuspended in 25 μL nuclear extraction buffer and vortexed for 5 min. Insoluble material was removed by centrifugation at 12,000× *g* for 10 min. The final nuclear protein fraction was transferred to a new prechilled microcentrifuge tube. Note that all steps involved in nuclear protein extraction were performed at 4 °C. Purity of the nuclear fraction was confirmed by immunoblotting with an anti-Histone H3 antibody (Abcam, Cambridge, MA, USA) and an anti-PEPC antibody (Agrisera, Vännäs, Sweden).

### 4.7. HY5 Degradation Assay and Immunoblot Analysis

To confirm the time-dependent degradation of HY5 under ER stress, we pretreated 10-day-old WT and *cop1* mutant plants with Tm (5 μg/mL) for 0, 6, and 12 h. Additionally, to confirm the proteasome-mediated degradation of HY5, we first treated the plants with or without Tm and then treated them with or without 50 μM MG132. Total proteins were extracted using non-denaturing buffer containing 100 mM Tris-Cl (pH 7.5), 150 mM NaCl, 0.5% NP-40, 1 mM EDTA, 3 mM DTT, and protease inhibitor cocktail, and were then separated by SDS-PAGE. The abundance of HY5 was assessed by immunoblotting with rabbit anti-HY5 antibody (1:1000; Agrisera). The antigen protein was detected by chemiluminescence using an ECL-detecting reagent (Thermo Scientific, Rockford, IL, USA). Rubisco L band (RbcL) stained with Ponceau S (Sigma-Aldrich) was used as the loading control.

### 4.8. Total RNA Exraction and Semi-Quantitative Reverse Transcription PCR (sqRT-PCR) Analysis

Seedlings were harvested and frozen in liquid nitrogen. Total RNA was extracted from the frozen seedlings using the RNA extraction kit (Qiagen, Valencia, CA, USA). RNA concentration and purity were determined using the NanoDrop ND-1000 spectrophotometer (NanoDrop Technologies, Wilmington, DE, USA). To remove genomic DNA contamination, we treated 1 μg total RNA with RNase-free DNaseI. First-strand cDNA was synthesized using oligo(dT)_18_ primer and Revert Aid M-MuLV Reverse Transcriptase (Thermo Scientific), according to the manufacturer’s instructions. Then, sqRT-PCR reactions were set up using 1 μL of twofold diluted cDNA and sequence-specific primers (Table S1). PCR cycling conditions were as follows: 95 °C for 5 min, followed by 28 cycles at 95 °C for 30 s, 55 °C for 20 s, and 72 °C for 60 s. *Tubulin2* (*Tub2*) served as a control gene. PCR products were separated by electrophoresis on 1% agarose gels and then stained with ethidium bromide.

### 4.9. Gene Expression Analysis by qRT-PCR

To examine gene expression, we performed qRT-PCR on a CFX 384 Touch™ Real-Time PCR Detection System (Bio-Rad Laboratories, Inc., Hercules, CA, USA) using a TOPreal™ qPCR 2X PreMIX (SYBR Green with high ROX) kit (Enzynomics, Daejeon, Korea). Target genes were amplified using sequence-specific primers (Table S1). Experiments were repeated at least three times, and expression levels were normalized against *ACT2*, *UBQ1*, and *UBQ10*.

### 4.10. ChIP Assay

ChIP experiments were performed as described previously using 2-week-old WT, *cop1-4*, and *hy5* seedlings grown in the presence or absence of Tm for 6 h. After harvesting, tissue samples (≈ 3 g) were crosslinked with 1% formaldehyde in a vacuum. The samples were ground to a powder in liquid nitrogen, and chromatin complexes were isolated and sonicated. Protein–DNA complexes were pulled down using protein-A agarose beads blocked with salmon sperm DNA (Upstate Biotechnology, Inc., Lake Placid, NY, USA), and anti-HY5 and anti-bZIP28 antibodies. Rabbit immunoglobulin G serum was used as a negative control. The anti-bZIP28 antibody was raised against a synthetic peptide corresponding to the amino acid sequence RSGDGGLEGRSE of the Arabidopsis bZIP28 protein (AT3G10800) (Abclone, Seoul, Korea). Relative enrichment of DNA was calculated by normalizing the amount of target DNA first to the internal control (*18s rRNA* gene) and then to the corresponding amount of the input DNA. The primers used for qPCR are listed in Table S1.

## Data Availability

Not applicable.

## Supplementary Materials

The following are available online at https://www.mdpi.com/article/10.3390/ijms221910772/s1.

## References

[1] Smith H. (2000). Phytochromes and light signal perception. Nature.

[2] Lin C. (2002). Blue light receptors and signal transduction. Plant Cell.

[3] Rizzini L., Favory J.J., Cloix C., Faggionato D., O’Hara A., Kaiserli E., Baumeister R., Schäfer E., Nagy F., Jenkins G.I. (2011). Perception of UV-B by the arabidopsis UVR8 protein. Science.

[4] Holm M., Deng X.W. (1999). Structural organization and interactions of COP1, a light-regulated developmental switch. Plant Mol. Biol..

[5] Ang L.H., Chattopadhyay S., Wei N., Oyama T., Okada K., Batschauer A., Deng X.W. (1998). Molecular interaction between COP1 and HY5 defines a regulatory switch for light control of Arabidopsis development. Mol. Cell.

[6] Oyama T., Shimura Y., Okada K. (1997). The Arabidopsis HY5 gene encodes a bZIP protein that regulates stimulus-induced development of root and hypocotyl. Genes Dev..

[7] Osterlund M.T., Hardtke C.S., Wei N., Deng X.W. (2000). Targeted destabilization of HY5 during light-regulated development of Arabidopsis. Science.

[8] Wang Q., Lin C. (2019). Photoreceptor signaling: When COP1 meets VPs. EMBO J..

[9] Podolec R., Ulm R. (2018). Photoreceptor-mediated regulation of the COP1/SPA E3 ubiquitin ligase. Curr. Opin. Plant Biol..

[10] Vitale A., Boston R.S. (2008). Endoplasmic reticulum quality control and the unfolded protein response: Insights from plants. Traffic.

[11] Liu J.X., Howell S.H. (2010). Endoplasmic reticulum protein quality control and its relationship to environmental stress responses in plants. Plant Cell.

[12] Beaugelin I., Chevalier A., D’Alessandro S., Ksas B., Havaux M. (2020). Endoplasmic reticulum-mediated unfolded protein response is an integral part of singlet oxygen signalling in plants. Plant J..

[13] Angelos E., Ruberti C., Kim S.J., Brandizzi F. (2017). Maintaining the factory: The roles of the unfolded protein response in cellular homeostasis in plants. Plant J..

[14] Srivastava R., Deng Y., Shah S., Rao A.G., Howell S.H. (2013). Binding protein is a master regulator of the endoplasmic reticulum stress sensor/transducer bZIP28 in Arabidopsis. Plant Cell.

[15] Iwata Y., Ashida M., Hasegawa C., Tabara K., Mishiba K.I., Koizumi N. (2017). Activation of the Arabidopsis membrane-bound transcription factor bZIP28 is mediated by site-2 protease, but not site-1 protease. Plant J..

[16] Liu J.X., Howell S.H. (2010). bZIP28 and NF-Y transcription factors are activated by ER stress and assemble into a transcriptional complex to regulate stress response genes in Arabidopsis. Plant Cell.

[17] Park J.H., Kang C.H., Nawkar G.M., Lee E.S., Paeng S.K., Chae H.B., Chi Y.H., Kim W.Y., Yun D.J., Lee S.Y. (2018). EMR, a cytosolic-abundant ring finger E3 ligase, mediates ER-associated protein degradation in Arabidopsis. New Phytol..

[18] Williams B., Verchot J., Dickman M.B. (2014). When supply does not meet demand-ER stress and plant programmed cell death. Front. Plant Sci..

[19] Xu G., Wang S., Han S., Xie K., Wang Y., Li J., Liu Y. (2017). Plant Bax Inhibitor-1 interacts with ATG6 to regulate autophagy and programmed cell death. Autophagy.

[20] Nawkar G.M., Kang C.H., Maibam P., Park J.H., Jung Y.J., Chae H.B., Chi Y.H., Jung I.J., Kim W.Y., Yun D.J. (2017). HY5, a positive regulator of light signaling, negatively controls the unfolded protein response in Arabidopsis. Proc. Natl. Acad. Sci. USA.

[21] McNellis T.W., von Arnim A.G., Wang D.X. (1994). Overexpression of Arabidopsis COP1 results in partial suppression of light-mediated development: Evidence for a light-inactivable repressor of photomorphogenesis. Plant Cell.

[22] Deng X.W., Quail P.H. (1992). Genetic and phenotypic characterization of cop1 mutants of Arabidopsis thaliana. Plant J..

[23] McNellis T.W., von Arnim A.G., Araki T., Komeda Y., Misera S., Wang D.X. (1994). Genetic and molecular analysis of an allelic series of cop1 mutants suggests functional roles for the multiple protein domains. Plant Cell.

[24] Chi Y.H., Melencion S.M.B., Alinapon C.V., Kim M.J., Lee E.S., Paeng S.K., Park J.H., Nawkar G.M., Jung Y.J., Chae H.B. (2017). The membrane-tethered NAC transcription factor, AtNTL7, contributes to ER-stress resistance in Arabidopsis. Biochem. Biophys. Res. Commun..

[25] Subramanian C., Kim B.H., Lyssenko N.N., Xu X., Johnson C.H., Von Arnim A.G. (2004). The Arabidopsis repressor of light signaling, COP1, is regulated by nuclear exclusion: Mutational analysis by bioluminescence resonance energy transfer. Proc. Natl. Acad. Sci. USA.

[26] Osterlund M.T., Deng X.W. (1998). Multiple photoreceptors mediate the light-induced reduction of GUS-COP1 from Arabidopsis hypocotyl nuclei. Plant J..

[27] Yu Y., Wang J., Shi H., Gu J., Dong J., Deng X.W., Huang R. (2016). Salt stress and ethylene antagonistically regulate nucleocytoplasmic partitioning of COP1 to control seed germination. Plant Physiol..

[28] Catalá R., Medina J., Salinas J. (2011). Integration of low temperature and light signaling during cold acclimation response in Arabidopsis. Proc. Natl. Acad. Sci. U.S.A..

[29] Karayekov E., Sellaro R., Legris M., Yanovsky M.J., Casal J.J. (2013). Heat shock-induced fluctuations in clock and light signaling enhance phytochrome B-mediated arabidopsis deetiolation. Plant Cell.

[30] Lee B.D., Kim M.R., Kang M.Y., Cha J.Y., Han S.H., Nawkar G.M., Sakuraba Y., Lee S.Y., Imaizumi T., McClung C.R. (2017). The F-box protein FKF1 inhibits dimerization of COP1 in the control of photoperiodic flowering. Nat. Commun..

[31] Stacey M.G., Kopp O.R., Kim T.H., Von Arnim A.G. (2000). Modular domain structure of Arabidopsis COP1. Reconstitution of activity by fragment complementation and mutational analysis of a nuclear localization signal in planta. Plant Physiol..

[32] Stacey M.G., Hicks S.N., Von Arnim A.G. (1999). Discrete domains mediate the light-responsive nuclear and cytoplasmic localization of Arabidopsis COP1. Plant Cell.

[33] Torii K.U., McNellis T.W., Deng X.W. (1998). Functional dissection of Arabidopsis COP1 reveals specific roles of its three structural modules in light control of seedling development. EMBO J..

[34] Ranjan A., Dickopf S., Ullrich K.K., Rensing S.A., Hoecker U. (2014). Functional analysis of COP1 and SPA orthologs from Physcomitrella and rice during photomorphogenesis of transgenic Arabidopsis reveals distinct evolutionary conservation. BMC Plant Biol..

[35] Lee J., He K., Stolc V., Lee H., Figueroa P., Gao Y., Tongprasit W., Zhao H., Lee I., Xing W.D. (2007). Analysis of transcription factor HY5 genomic binding sites revealed its hierarchical role in light regulation of development. Plant Cell.

[36] Zhang H., He H., Wang X., Wang X., Yang X., Li L., Deng X.W. (2011). Genome-wide mapping of the HY5-mediated genenetworks in Arabidopsis that involve both transcriptional and post-transcriptional regulation. Plant J..

[37] Yamaguchi N., Winter C.M., Wu M.-F., Kwon C.S., William D.A., Wagner D. (2014). PROTOCOL: Chromatin Immunoprecipitation from Arabidopsis Tissues. Arab. B..

[38] Nawkar G.M., Lee E.S., Shelake R.M., Park J.H., Ryu S.W., Kang C.H., Lee S.Y. (2018). Activation of the transducers of unfolded protein response in plants. Front. Plant Sci..

[39] Nieto C., Luengo L.M., Prat S. (2020). Regulation of COP1 Function by Brassinosteroid Signaling. Front. Plant Sci..

[40] Zhang X., Huai J., Shang F., Xu G., Tang W., Jing Y., Lin R. (2017). A PIF1/PIF3-HY5-BBX23 transcription factor cascade affects photomorphogenesis. Plant Physiol..

[41] Ponnu J., Hoecker U. (2021). Illuminating the COP1/SPA Ubiquitin Ligase: Fresh Insights Into Its Structure and Functions During Plant Photomorphogenesis. Front. Plant Sci..

[42] Alabadí D., Gallego-Bartolomé J., Orlando L., García-Cárcel L., Rubio V., Martínez C., Frigerio M., Iglesias-Pedraz J.M., Espinosa A., Deng X.W. (2008). Gibberellins modulate light signaling pathways to prevent Arabidopsis seedling de-etiolation in darkness. Plant J..

[43] Vandenbussche F., Habricot Y., Condiff A.S., Maldiney R., Van Der Straeten D., Ahmad M. (2007). HY5 is a point of convergence between cryptochrome and cytokinin signalling pathways in Arabidopsis thaliana. Plant J..

[44] Wang W., Paik I., Kim J., Hou X., Sung S., Huq E. (2021). Direct phosphorylation of HY5 by SPA kinases to regulate photomorphogenesis in Arabidopsis. New Phytol..

[45] Gangappa S.N., Holm M., Botto J.F. (2013). Molecular interactions of BBX24 and BBX25 with HYH, HY5 HOMOLOG, to modulate Arabidopsis seedling development. Plant Signal. Behav..

[46] Datta S., Hettiarachchi C., Johansson H., Holm M. (2007). Salt Tolerance Homolog2, a B-box protein in Arabidopsis that activates transcription and positively regulates light-mediated development. Plant Cell.

[47] Holtan H.E., Bandong S., Marion C.M., Adam L., Tiwari S., Shen Y., Maloof J.N., Maszle D.R., Ohto M., Preuss S. (2011). Bbx32, an arabidopsis b-box protein, functions in light signaling by suppressing HY5-regulated gene expression and interacting with STH2/BBX21. Plant Physiol..

[48] Jeong R.D., Chandra-Shekara A.C., Barman S.R., Navarre D., Klessig D.F., Kachroo A., Kachroo P. (2010). Cryptochrome 2 and phototropin 2 regulate resistance protein-mediated viral defense by negatively regulating an E3 ubiquitin ligase. Proc. Natl. Acad. Sci. USA.

[49] Kim J.Y., Jang I.C., Seo H.S. (2016). COP1 controls abiotic stress responses by modulating AtSIZ1 function through its E3 ubiquitin ligase activity. Front. Plant Sci..

[50] Lin X.L., Niu D., Hu Z.L., Kim D.H., Jin Y.H., Cai B., Liu P., Miura K., Yun D.J., Kim W.Y. (2016). An Arabidopsis SUMO E3 Ligase, SIZ1, Negatively Regulates Photomorphogenesis by Promoting COP1 Activity. PLoS Genet..

[51] Mishiba K.I., Nagashima Y., Suzukia E., Hayashi N., Ogata Y., Shimada Y., Koizumi N. (2013). Defects in IRE1 enhance cell death and fail to degrade mRNAs encoding secretory pathway proteins in the Arabidopsis unfolded protein response. Proc. Natl. Acad. Sci. USA.

[52] Kang C.H., Jung W.Y., Kang Y.H., Kim J.Y., Kim D.G., Jeong J.C., Baek D.W., Jin J.B., Lee J.Y., Kim M.O. (2006). AtBAG6, a novel calmodulin-binding protein, induces programmed cell death in yeast and plants. Cell Death Differ..

[53] Curtis M.D., Grossniklaus U. (2003). A Gateway Cloning Vector Set for High-Throughput Functional Analysis of Genes in Planta. Plant Physiol..

[54] Zhang X., Henriques R., Lin S.S., Niu Q.W., Chua N.H. (2006). Agrobacterium-mediated transformation of Arabidopsis thaliana using the floral dip method. Nat. Protoc..

