# Supplementary material for: Constitutive Photomorphogenic 1 Enhances ER Stress Tolerance in Arabidopsis
Source: Int J Mol Sci. 2021 Oct 5;22(19):10772. doi: 10.3390/ijms221910772 (PMC8509555; doi:10.3390/ijms221910772)
Supplement: Supplementary file 1 [file ijms-22-10772-s001.zip › ijms-1343914-supplementary.pdf]

**Supplementary Materials**

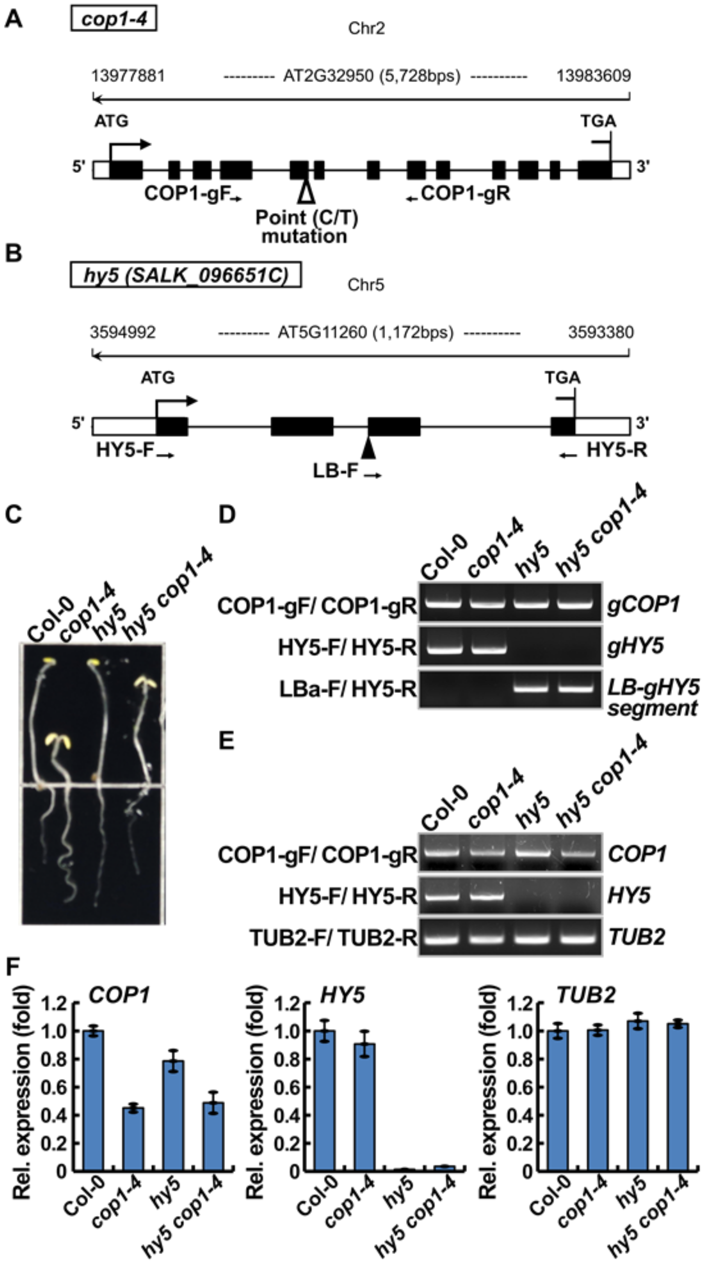


**Figure S1.** Identification of *cop1-4*, *hy5*, and *hy5 cop1-4* mutants. (**a, b**) Schematic representations of the predicted gene structures of *HY5* (**a**) and *COP1* (**b**). Black elbow arrows represent the start codon (ATG). Filled boxes and horizontal black lines indicate exons and introns, respectively. Blank boxes at either end of the genes represent 5′ and 3′ untranslated regions (UTRs). The position of T-DNA insertion in *hy5* and that of the point mutation in *cop1-4* are indicated by black and white arrowheads, respectively. Black arrows represent the binding sites of primers used for genotyping. (**c–f**) Confirmation of *cop1-4*, *hy5* and *hy5 cop1-4* mutant lines by phenotypes of 5-day-dark-grown plants (**c**), genomic DNA-based PCR (**d**), semi-quantitative reverse transcription PCR (sqRT-PCR), (**d**) and quantitative real-time PCR (qRT-PCR) (**f**). (**f**) Expression analysis of *COP1*, *HY5*, and *TUB2* (control) genes in 2-week-old WT (Col-0), *cop1-4*, *hy5*, and *hy5 cop1-4* plants by qRT-PCR. Data represent mean ± standard error of mean (SEM).

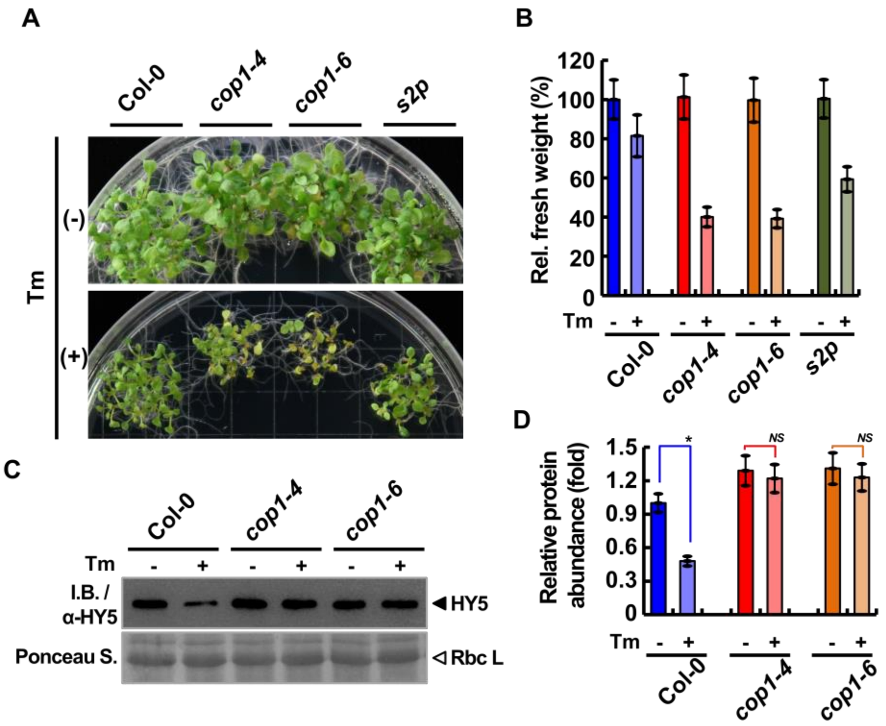


**Figure S2.** Confirmation of the role of COP1 in ER stress response using the *cop1-6* mutant. (**a**) Phenotypes of 2-week-old WT (Col-0), *cop1-4*, *cop1-6*, and *s2p* seedlings grown on Murashige and Skoog (MS) medium supplemented with (+) or without (-) 10 ng/mL tunicamycin (Tm). (**b**) Relative fresh weight of plants treated with Tm, as indicated in (**a**). Data represent mean ± SD (*n* = 3). (**c**) Comparison of Tm-induced changes in the amount of HY5 protein in WT (Col-0), *cop1-4*, and *cop1-6* seedlings. Total proteins extracted from 10-day-old seedlings before (-) or after (+) 6 h treatment with 5 μg/mL Tm were subjected to immunoblotting analysis with anti-HY5 antibody. (**d**) Quantification of the relative abundance of HY5 protein in (c) compared with that in the control condition (Col-0, -).

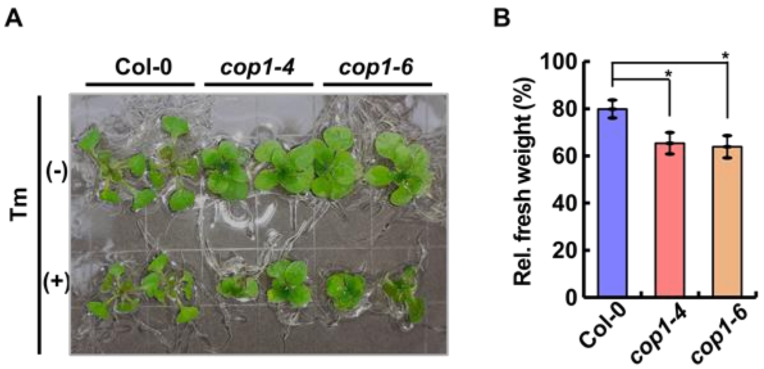


**Figure S3.** Comparison of growth of WT (Col-0) and *cop1* mutant plants after treatment of 5 μg/mL Tm for 6 h. 10-day-old WT, *cop1-4*, and *cop1-6* plantlets were treated 5 μg/mL Tm for 6 h, and allowed to recover on MS plates. (**a**) Plants were analyzed for phenotypic differences 5 days post treatment. (**b**) Relative fresh weight of plants treated with Tm (+) compared to the Tm-untreated plants (-), as indicated in (**a**). Data represent mean ± SD ($n$ = 3).

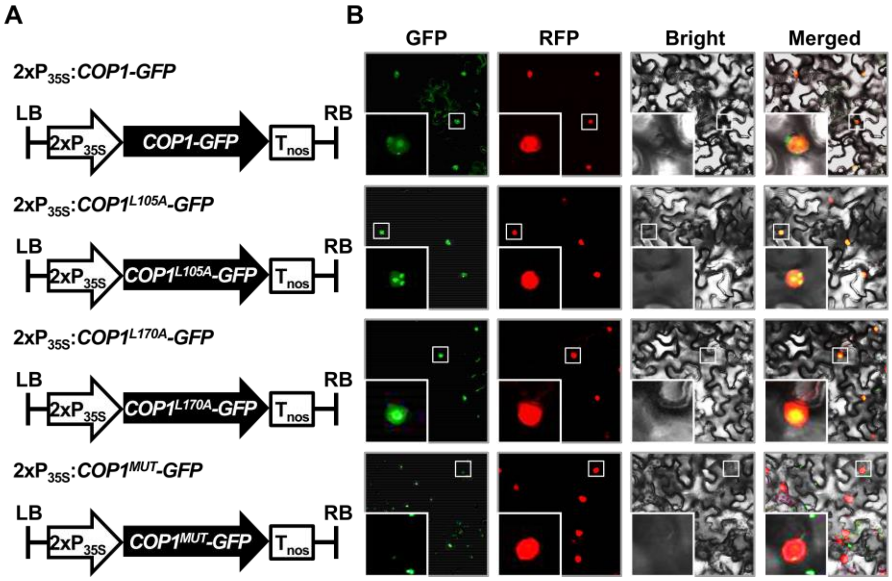


**Figure S4.** Subcellular localization analysis of WT and mutant COP1 proteins. (**a**) Schematic representations of constructs containing diverse *COP1* alleles. $P_{35S}$ and $T_{NOS}$ represent the cauliflower mosaic virus (CaMV) *35S* promoter, and *nopaline synthase* (*NOS*) terminator, respectively. (**b**) Subcellular localization analysis of green fluorescent protein (GFP)-fused diverse COP1 proteins in *Agrobacterium*-infiltrated tobacco leaves. Tobacco leaves were infiltrated with *Agrobacteria* harboring diverse *COP1* allele constructs depicted in (**a**). At 2 days post-infiltration, the localization of GFP-fused diverse COP1 proteins was observed under a confocal microscope. Green color indicates GFP-fused diverse COP1 proteins, whereas red color indicates NLS-RFP (nucleus marker). Inset images represent f-fold magnification of the small squares outlined with white lines in the GFP panel.

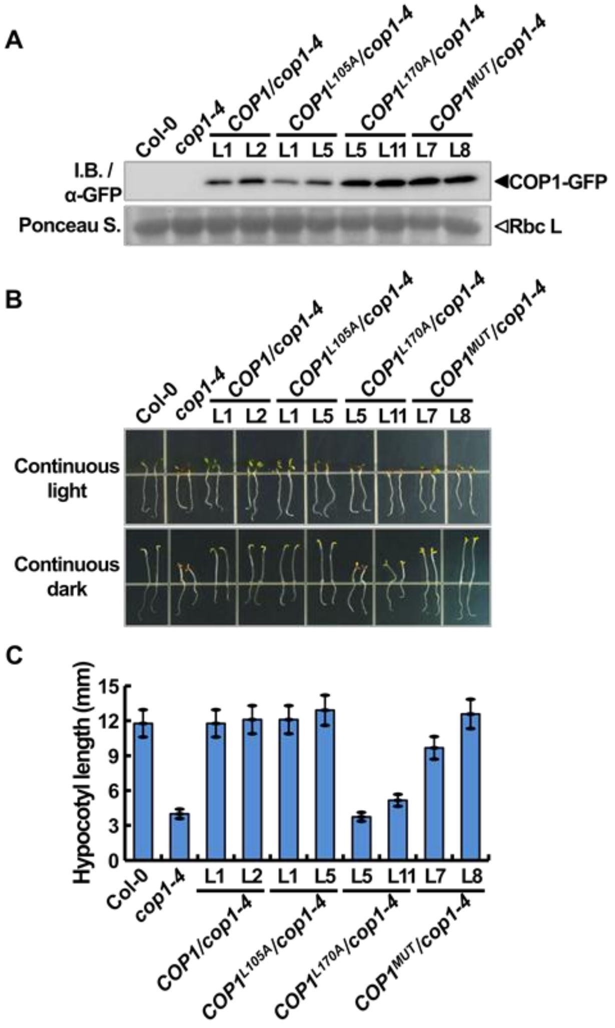


**Figure S5.** Selection of *cop1-4* complementation lines expressing *COP1*, *COP1*$^{L105A}$, *COP1*$^{L170A}$, and *COP1*$^{MUT}$. (**a**) Detection of GFP-fused COP1 proteins in selected *cop1-4* complementation lines transformed with *COP1*, *COP1*$^{L105A}$, *COP1*$^{L170A}$, and *COP1*$^{MUT}$ constructs. Total proteins extracted from 2-week-old seedlings of the WT (Col-0), *cop1-4* mutant, and selected *cop1-4* complementation lines were subjected to immunoblotting analysis using anti-GFP antibody. (**b**) Photographs of seedlings of different genotypes grown for 5 days under either continuous light or continuous dark conditions. (**c**) Hypocotyl lengths of seedlings grown under continuous dark. Data represent mean ± SD ($n$ = 3).

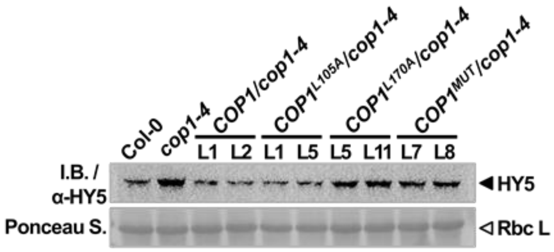


**Figure S6.** Comparison the amount of HY5 protein in Tm-treated seedlings of WT (Col-0), *cop1-4*, and various *cop1-4* complementation lines. Total proteins extracted from 10-day-old seedlings after 6 h treatment with 5 μg/mL Tm were subjected to immunoblotting analysis with anti-HY5 antibody.

**Table S1. List of primers used in this study.**

| Gene | AGI code | Primer name | Sequence (5'-3') | Purpose |
|---|---|---|---|---|
| *COP1* | AT2G32950 | COP1 qF | GAGGCAGGAAGCAAGTGTGA | qRT-PCR |
| | | COP1 qR | CGACCGCAATGTAGTTGCTT | |
| *HY5* | AT5G11260 | HY5 qF | CACTACAGCCGGTATGCAAG | |
| | | HY5 qR | CGATCCTAAACCAAACCCTTC | |
| *TUB2* | AT5G62690 | TUB2 qF | AAACTCACTACCCCCAGCTTTG | |
| | | TUB2 qR | CACCAGACATAGTAGCAGAAATCAAGT | |
| *ACT2* | AT3G18780 | ACT2 qF | TGATGCACTTGTGTGTGACAA | |
| | | ACT2 qR | GGGACTAAAACGCAAAACGA | |
| *UBQ1* | At3g52590 | UBQ1 qF | TTCCTTGATGATGCTTGCTC | |
| | | UBQ1 qR | TTGACAGCTCTTGGGTGAAG | |
| *UBQ10* | AT4G05320 | UBQ10 qF | AGATCCAGGACAAGGAGGTATTC | |
| | | UBQ10 qR | CGCAGGACCAAGTGAAGAGTAG | |
| *BIP3* | AT1G09080 | BIP3 CHIP-1F | GATTTAATGTCACGTGTCTGCTTGT | ChIP |
| | | BIP3 CHIP-1R | TTGGCGCGCTCCTTACTT | |
| | | BIP3 CHIP-2F | CAAAATAACCCATTAAGCTTACGTG | |
| | | BIP3 CHIP-2R | CTCGGTAGAGTGTCCTCTCCAAT | |
| *TA3 retrotransposon* | AT1G37110 | TA3 FW | CTGCGTGGAAGTCTGTCAAA | |
| | | TA3 RW | CTATGCCACAGGGCAGTTTT | |
| *18s rRNA* | AT3G41768 | 18s rRNA qF | GGGCATTCGTATTTCATAGT | |
| | | 18s rRNA qR | CGTTCTTGATTAATGAAAAC | |
| *COP1* | AT2G32950 | COP1 gF | CTCTCATGGGCTACCAAAGA | Geno-typing |
| | | COP1 gR | TACATCCCACACTGTTACTA | |
| *HY5* | AT5G11260 | HY5-F | ATGCAGGAACAAGCGACTAG | |
| | | HY5-R | TCAAAGGCTTGCATCAGCAT | |
| *TUB2* | AT5G62690 | TUB-F | CCAACAACGTGAAATCGACA | |
| | | TUB-R | TCTTGGTATTGCTGGTACTC | |
| Lba-T-DNA | | LB-F | TGGTTCACGTAGTGGGCCATCG | |
| *COP1* | AT2G32950 | COP1 F | ATGGAAGAGATTTCGACGGAT | Cloning |
| | | COP1 R | TCACGCAGCGAGTACCAGAAC | |
| | | COP1 attB1 F * | *AAAAAGCAGGCCAT*ATGGAAGAGATTTCGACG | |
| | | COP1 attB2 R * | *AGAAAGCTGGGT*TCACGCAGCGAGTACCAG | |
| attB | | attB1 * | *GGGGACAAGTTTGTACAAAAAAGCAGGCCAT* | |
| | | attB2 * | *GGGGACCACTTTGTACAAGAAAGCTGGGT* | |
| $P_{BIP3}$:*LUC* (Reporter construct) | AT1G09080 | LUC($P_{BIP3}$)-F | CGAAAAATGGAAGACGCCAAAAACATAAAG | |
| | | LUC($T_{35S}$)-R | CTCGAGATCTGGATTTTAGTACTGGATTTT | |
| | | $P_{BIP3}$-F | AGCTGGAGCTCGCATTCACTACAATTACAT | |
| | | $P_{BIP3}$-R | CGTCTTCCATTTTTCGTTGTTGAGAACTCT | |
| | | $P_{BIP3}$-FE | CTAAAGGGAACAAAAGCTGGAGCTCGCATT | |
| | | LUC($T_{35S}$)-RE | CCGGGCCCCCCCTCGAGATCTGGATTTTAG | |
| *COP1* | AT2G32950 | COP1(L105A) F | GCGGCCGATAAGGCAGCGAAGAAAACTTCAGCT | Site-directed mutation |
| | | COP1(L105A) R | TTCGCTGCCTTATCGGCCGCGAAATTAGGGTAA | |
| | | COP1(L170A) F | GCTGCGGACTTTGCGCATTGTGCAAGGAAGCAA | |
| | | COP1(L170A) R | GCACAATGCGCAAAGTCCGCAGCTATCTGCATG | |
| | | COP1(MUT1) F | TCTCAATGGCTAGTAAGACACGGATTCATGCTC | |
| | | COP1(MUT1) R | GAGCATGAATCCGTGTCTTACTAGCCATTGAGA | |
| | | COP1(MUT2) F | ATGTTACCTCCAAACGCGGAGTCAGTTGGCAGA | |
| | | COP1(MUT2) R | TCTGCCAACTGACTCCGCGTTTGGAGGTAACAT | |

- attB1 and attB2 sites (*) are italicized.
